# Dopamine Receptor and Gα(olf) Expression in DYT1 Dystonia Mouse Models during Postnatal Development

**DOI:** 10.1371/journal.pone.0123104

**Published:** 2015-04-10

**Authors:** Lin Zhang, Deirdre M. McCarthy, Nutan Sharma, Pradeep G. Bhide

**Affiliations:** 1 Department of Biomedical Sciences, Center for Brain Repair, Florida State University College of Medicine, Tallahassee, Florida, United States of America; 2 Neurology, Massachusetts General Hospital and Harvard Medical School, Boston, Massachusetts, United States of America; University of Chicago, UNITED STATES

## Abstract

**Background:**

DYT1 dystonia is a heritable, early-onset generalized movement disorder caused by a GAG deletion (ΔGAG) in the *DYT1* gene. Neuroimaging studies and studies using mouse models suggest that DYT1 dystonia is associated with dopamine imbalance. However, whether dopamine imbalance is key to DYT1 or other forms of dystonia continues to be debated.

**Methodology/Principal Findings:**

We used *Dyt1* knock out (*Dyt1* KO), *Dyt1* ΔGAG knock-in (*Dyt1* KI), and transgenic mice carrying one copy of the human *DYT1* wild type allele (*DYT1 *hWT) or human ΔGAG mutant allele (*DYT1 *hMT). D1R, D2R, and Gα(olf) protein expression was analyzed by western blot in the frontal cortex, caudate-putamen and ventral midbrain in young adult (postnatal day 60; P60) male mice from all four lines; and in the frontal cortex and caudate putamen in juvenile (postnatal day 14; P14) male mice from the *Dyt1* KI and KO lines. Dopamine receptor and Gα(olf) protein expression were significantly decreased in multiple brain regions of *Dyt1* KI and *Dyt1* KO mice and not significantly altered in the *DYT1 *hMT or *DYT1 *hWT mice at P60. The only significant change at P14 was a decrease in D1R expression in the caudate-putamen of the *Dyt1 *KO mice.

**Conclusion/Significance:**

We found significant decreases in key proteins in the dopaminergic system in multiple brain regions of *Dyt1* KO and *Dyt1* KI mouse lines at P60. Deletion of one copy of the *Dyt1* gene (KO mice) produced the most pronounced effects. These data offer evidence that impaired dopamine receptor signaling may be an early and significant contributor to DYT1 dystonia pathophysiology.

## Introduction

Dystonia is the third most common movement disorder after essential tremor and Parkinson’s disease. It is a neurological disorder characterized by involuntary muscle contractions with debilitating, painful, twisting movements and contorted postures [1]. Although the vast majority of dystonia is sporadic, it can be inherited or may be a consequence of traumatic or vascular brain injury or side effect of medications [2,3].

DYT1 dystonia is an inherited form of early onset generalized dystonia. It is inherited in an autosomal dominant manner, and has relatively low penetrance [4,5]. A trinucleotide deletion in the *TOR1A* gene, which results in the loss of a glutamic acid residue in the C-terminus region of the torsinA protein is linked to DYT1 dystonia [6]. TorsinA is a member of AAA+ ATPase superfamily [6], associated with chaperone like functions in multiple processes including protein folding and degradation, cytoskeletal dynamics, membrane trafficking, vesicle fusion and transportation and secretion [7,8]. TorsinA mRNA is expressed in dopaminergic neurons of the substantia nigra pars compacta, granule and pyramidal neurons of the hippocampus, Purkinje and granule neurons of the cerebellum, and cholinergic neurons of the neostriatum in humans [9,10].

Although the pathophysiological basis of dystonia remains elusive, it is believed to involve dysfunction of motor circuits in the cerebral cortex, thalamus, cerebellum and basal ganglia [11–13]. In particular, the striatal dopaminergic system is considered to be involved [13]. Reduced striatal D2R binding was reported in humans with various forms of dystonia including DYT1 dystonia, idiopathic cervical dystonia, and nocturnal myoclonus [14–16]. In addition, decreased dopamine release was reported in the *DYT1* hMT transgenic mice [17].

Recently mutations in the gene for guanine nucleotide binding protein alpha subunit [Gα(olf)], were found to be associated with primary dystonias, including some cases of early onset generalized dystonia [18]. Gα(olf) is enriched in the striatum, and it forms heterotrimeric complex with Gβ/Gγ7 [19]. These second messengers are coupled with dopamine D1 receptors, suggesting further involvement of the dopaminergic system in another form of dystonia.

Despite the compelling evidence described above, involvement of the dopaminergic system in dystonia remains a subject of debate. Moreover, whether changes in dopaminergic signaling begin early in development is also not clear. Here, we examined D1R, D2R and Gα(olf) protein expression in the frontal cortex, caudate putamen and ventral midbrain in four lines of DYT1 dystonia mouse models. We reasoned that a finding of dopaminergic dysfunction in multiple models of DYT1 dystonia could add additional support for involvement of the dopaminergic system in this form of dystonia.

## Materials and Methods

### Animals

The following mouse models were used: Heterozygous *Dyt1* knock-out (*Dyt1* KO), *Dyt1* heterozygous ΔGAG knock-in (*Dyt1* KI), hemizygous human *DYT1* wild type transgenic *(DYT1* hWT) and hemizygous human *DYT1* ΔGAG transgeni*c (DYT1* hMT). The generation of the mouse lines has been described previously [20–22]. Homozygous *Dyt1* KO and *Dyt1* KI mice die within 2–3 days of birth, and only heterozygous mice are viable. *DYT1* hWT and *DYT1* hMT mice are hemizygous for the respective transgene (i.e. only one allele bore the transgene), and survive to adulthood [21]. For each line we bred heterozygous or hemizygous mice with wild type partners to produce wild type and hetero/hemizygous offspring. Offspring were genotyped at the time of weaning using methods described previously [20–22]. We used wild type littermates from each line as controls for that line. Mice were kept in temperature and humidity controlled rooms under a 12-h-light/dark cycle with free access to food and water in the laboratory animal facility at the Massachusetts General Hospital, Boston, MA. The experimental studies were approved by the Massachusetts General Hospital’s Institutional Animal Care and Use Committee (IACUC). All of the experimental procedures were in full compliance with institutional guidelines at the Massachusetts General Hospital and the NIH *Guide for the Care and Use of Laboratory Animals*. The experiments were performed by investigators blinded to the genotypes.

### Western blot analysis

Postnatal day 14 (P14; day of birth = P0) and P60 male mice from each line along with wild type male littermate controls were decapitated (mice were euthanized by anesthetic overdose which is consistent with the recommendations of the Panel on Euthanasia of the American Veterinary Medical Association) and the brains removed. Frontal cortex, caudate-putamen and ventral midbrain were micro-dissected based on anatomical landmarks. Samples of each region from the two hemispheres of a given brain were pooled such that there was only one sample for each brain region from one mouse. Our expertise in microdissection and isolation of different brain regions of the adult and developing mice [23–28], helps assure reliable sampling of the brain regions. We were also concerned that volumetric changes in the brains of transgenic mice could contribute variability to the sampling procedure. However, to the best of our knowledge, volumetric changes in the caudate-putamen, frontal cortex or midbrain do not exist in any of the mouse lines used here [20,21]. The tissue was homogenized in 200 μl of ice-cold lysis buffer containing 50mM Tris-Cl (pH 7.4), 175mM NaCl, 5mM EDTA with a protease inhibitor cocktail tablet (Roche, 05892791001) and sonicated for 10 sec. Triton X-100 was added to 1% w/v final concentration and the mixture was incubated on ice for 30 min and centrifuged at 10,000 × g for 15 min at 4°C. The protein concentration was measured using the Bradford assay with bovine serum albumin (BSA; Fisher Scientific, A7906) standard. The homogenates were mixed with SDS-PAGE loading buffer and boiled for 5 min, followed by 1-minute incubation on ice and 5 minutes of gentle centrifugation at 8,000g. The supernatant was stored at -20°C till the day of analysis. 40 μg of each sample was loaded on a 10% SDS-PAGE and the separated proteins were transferred to PVDF membrane. After blocking with 5% BSA or 5% milk in TBS-T buffer, which contains 20mM Tris-Cl (pH 7.6), 137 mM NaCl, 0.1% Tween 20, the membranes were incubated overnight at 4°C with rabbit anti-torsinA antibody (1:500; Abcam, ab34540) in 5% milk TBS-T buffer, rabbit anti-D2R antibody (1:500; Millipore, AB5084P) in 5% BSA TBS-T buffer (Abcam, Cambridge, MA), rabbit anti-D1R antibody (1:200; Santa Cruz, sc-14001) in 3% BSA TBS-T buffer, rabbit anti-Gα(olf), antibody (1:500; Abcam, ab74049) in 5% BSA TBS-T buffer, or rabbit anti-Gα(S) antibody (1:500; Santa Cruz, sc-823) in 3% BSA TBS-T buffer [29–35]. Membranes were washed three times and incubated with bovine anti-rabbit IgG-HRP (1:15000; Santa Cruz, sc-2370) in 5% BSA TBS-T at room temperature for 1 hour. β-actin was used as a loading control, and the membranes were also probed with HRP-conjugated β-actin antibody (1:5000; Santa Cruz, sc-4778). Immunoreactive bands were detected using Super Signal West Pico Chemiluminescent Substrate (Thermo Scientific). The signals were captured by Alpha Innotech FluorChem FC2 and quantified with UN-SCAN-IT gel (Silk Scientific) software. Each western blot experiment was repeated three times.

### Statistical analysis

Intensity of each band from each western blot for each experimental group (i.e. wild type and transgenic) in each experiment was normalized to the intensity of the β-actin band (loading control) for that blot. Thus, expression of each protein [D1R, D2R or Gα(olf)] was analyzed in independent and separate blots for every brain region in every mouse line and at each age. Mean±SEM values of normalized band intensities were calculated for each protein by using 3–4 replications. Each brain sample was considered as one replication. Thus, we had 3 to 4 replications for each protein (n = 3 to 4). The data were analyzed using One-Way ANOVA, followed by Bonferroni post hoc analysis (Prism 6 Software). A *p*-value smaller than 0.05 was considered to be statistically significant.

## Results

### D1R expression

**Fig 1:**
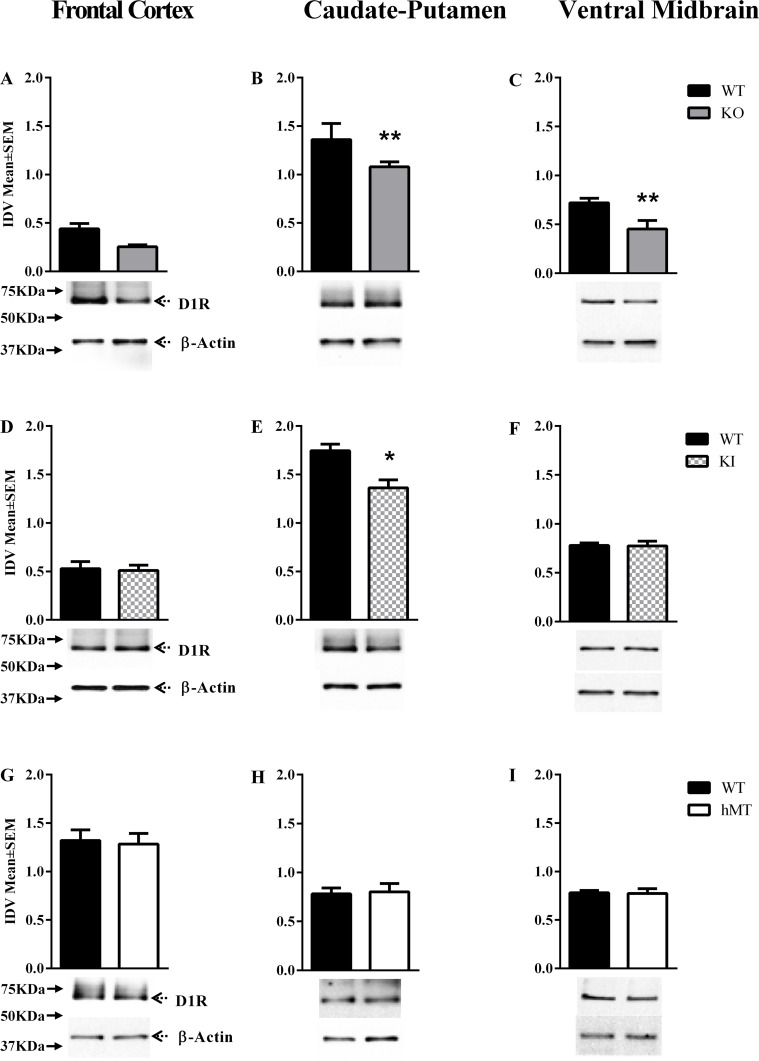
D1R expression at P60. D1R expression in the frontal cortex, caudate-putamen and ventral midbrain of postnatal day 60 (P60; A-I) mice. In each panel, the upper band shows D1R and the lower band shows β-actin, which was used as a loading control. The bar graphs in each panel represent D1R band intensity (mean ± SEM) normalized to intensity of the loading control (integrated density value; IDV). The names of the mouse lines (*Dyt1* KO, *Dyt1* KI, hMT) are indicated to the right. (*p<0.05, **p<0.01; n = 3 or 4).

**Fig 2:**
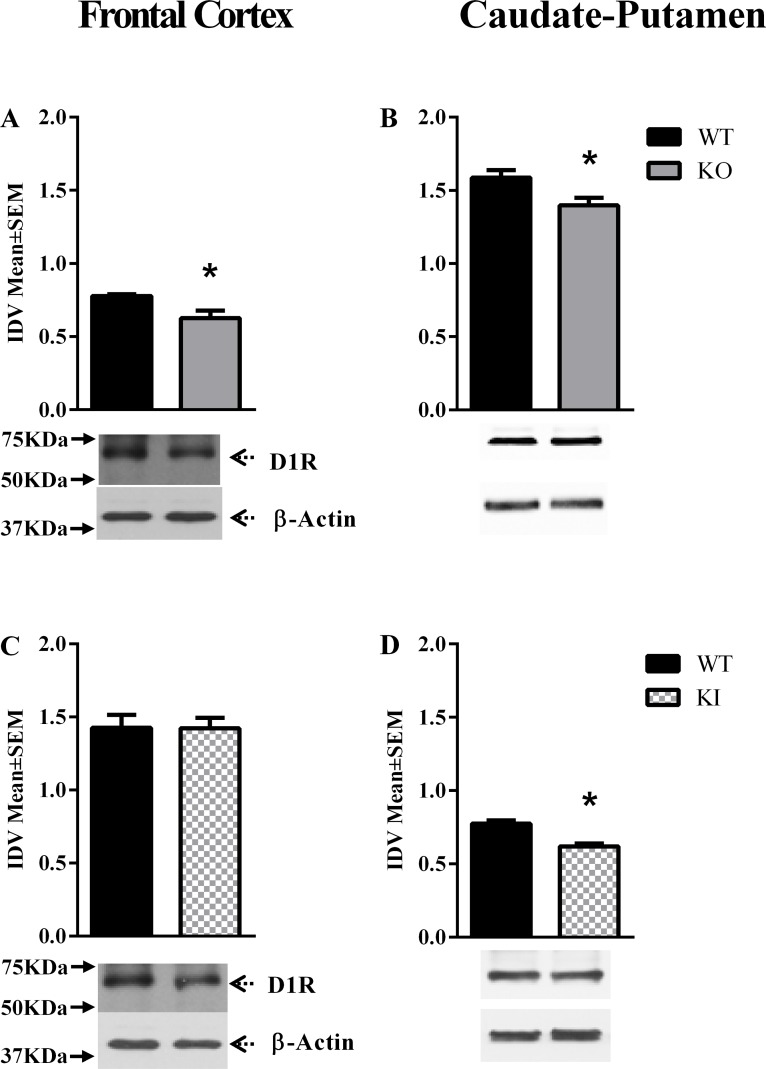
D1R expression at P14. D1R expression in the frontal cortex and caudate-putamen of postnatal day 14 (P14; A- D) mice. In each panel, the upper band shows D1R and the lower band shows β-actin, which was used as a loading control. The bar graphs in each panel represent D1R band intensity (mean ± SEM) normalized to intensity of the loading control (integrated density value; IDV). The names of the mouse lines (*Dyt1* KO, *Dyt1* KI) are indicated to the right. (*p<0.05, n = 3 or 4).

D1R expression in the frontal cortex, caudate putamen and ventral midbrain showed significant changes in the *Dyt1* KO [F = (5,12) = 63.88, P < 0.0001] and *Dyt1* KI [F = (5,17) = 61.15, P < 0.0001] lines of mice at P60 [Table 1]. Post hoc analysis revealed significant reductions in D1R expression in the caudate-putamen and ventral midbrain regions in the *Dyt1* KO mice [Mean±SEM; caudate-putamen: WT = 1.36±0.10, KO = 1.08±0.03, p = 0.0084; ventral midbrain: WT = 0.72±0.03, KO = 0.45±0.05, p = 0.012; Fig 1A–1C] and only in the caudate-putamen in the *Dyt1* KI mice [Mean±SEM; caudate-putamen: WT = 1.75±0.07, KI = 1.36±0.08, p = 0.002; Fig 1E]. D1R expression was not significantly altered in the *DYT1* hMT or hWT mice [Fig 1G–1I, and S1 Table]. D1R expression at P14 [Table 2] showed significant changes only in the *Dyt1* KO line [F = (3,12) = 107.4, P < 0.0001, Fig 2B]. Post-hoc analysis revealed significant reductions only in the caudate putamen of this mouse line [Mean±SEM; WT = 1.59±0.05, KO = 1.40±0.05, p = 0.023, Fig 2B and S4 Table]. We did not analyze *DYT1* hWT or hMT lines of mice at P14.

**Table 1:** A summary of the changes in D1R, D2R and Gα(olf) expression at P60.

	P60 D1R			P60 D2R			P60 Gα(olf)		
	FC	CP	vMB	FC	CP	vMB	FC	CP	vMB
**KO**	–	**↓**	**↓**	**↓**	**↓**	**↓**	**↓**	–	**↓**
**KI**	–	**↓**	–	**↓**	–	–	–	–	–
**hWT**	–	–	–	–	–	–	N/A	N/A	N/A
**hMT**	–	–	–	–	–	–	N/A	N/A	N/A

A summary of the changes in D1R, D2R and Gα(olf) expression at postnatal day 60 (P60) in the frontal cortex (FC), caudate-putamen (CP) and ventral midbrain (vMB) in the Dyt1 KO (KO), Dyt1 KI (KI), human wild-type torsinA (hWT) and human mutant torsinA (hMT) lines of mouse.

**↓** indicates statistically significant reductions in expression compared to wild-type control mice.

—indicates no statistically significant difference.

N/A differences were not analyzed.

**Table 2:** A summary of the changes in D1R, D2R and Gα(olf) expression at P14.

	D1R		D2R		Gα(olf)	
	FC	CP	FC	CP	FC	CP
**KO**	–	**↓**	–	–	–	–
**KI**	–	–	–	–	–	–

A summary of the changes in D1R, D2R and Gα(olf) expression at postnatal day 14 (P14) in the frontal cortex (FC) and caudate-putamen (CP) in the Dyt1 KO (KO) and Dyt1 KI (KI) lines of mouse.

**↓** indicates statistically significant reductions in expression compared to wild-type control mice.

—indicates no statistically significant difference.

### Gα(olf) expression

**Fig 3:**
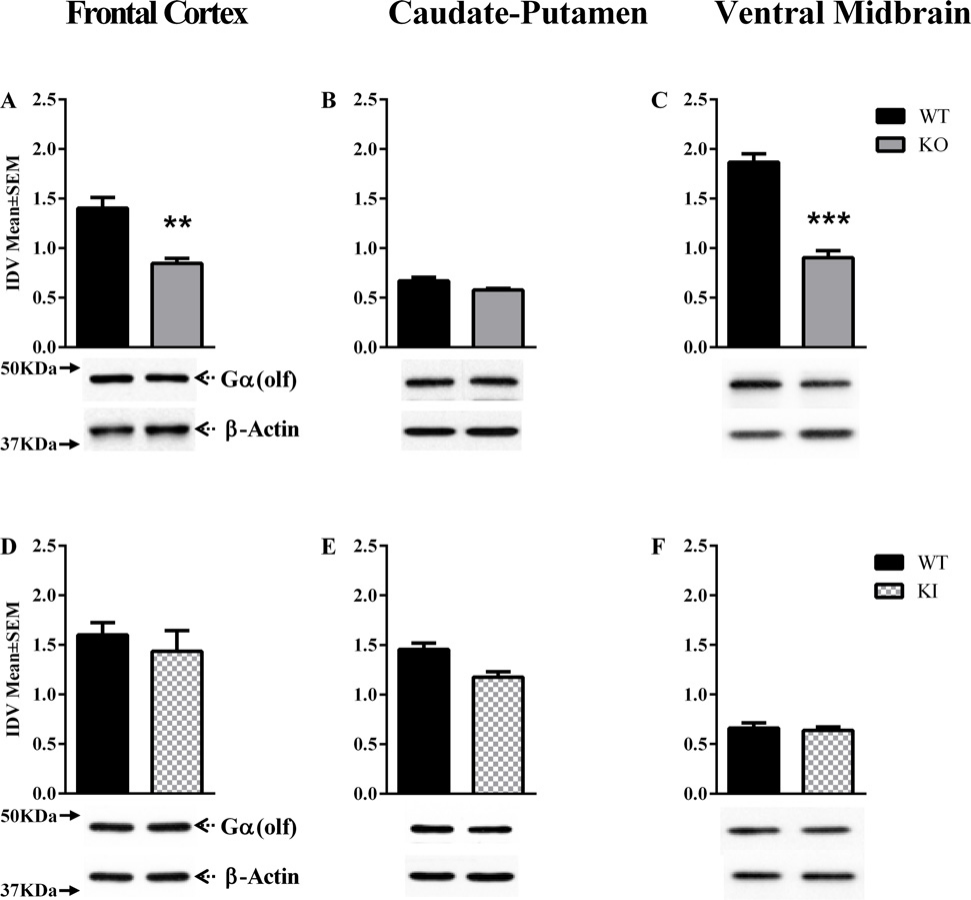
Gα(olf) and Gα(s) expression at P60. Gα(olf) and Gα(s) expression in the frontal cortex, caudate-putamen and ventral midbrain of Dyt1 KO, and, Dyt1 KI mice at postnatal day 60 (P60; A-F). In each panel, the upper band shows Gα(olf) (A-F), and the lower band shows β-actin, which was used as a loading control. The bar graphs in each panel represent Gα(olf) band intensity (mean ± SEM) normalized to intensity of loading control (integrated density value; IDV). The names of the mouse lines (*Dyt1* KO, *Dyt1* KI) are indicated to the right (**p<0.01, ***p<0.001; n = 3 or 4).

Only the *Dyt1* KO mice showed significant changes in Gα(olf) expression at P60 [F = (5,12) = 51.60, P < 0.0001, Table 1]. Post hoc analysis indicated a significant reduction in the frontal cortex and ventral midbrain [Mean± SEM; frontal cortex: WT = 1.40±0.85, KO = 0.84±0.05, p = 0.0003; ventral midbrain: WT = 1.87±0.08, KO = 0.90±0.07, p = 0.0001; Fig 3A–3C]. Gα(olf) levels did not show significant changes in the *Dyt1* KI, *DYT1* hMT or hWT mice [S2 Table]. We examined Gα(olf) expression in the *Dyt1* KI and KO mice at P14 [Table 2]. However, neither line of mouse showed significant changes in any of the brain regions examined [S5 Table].

**Fig 4:**
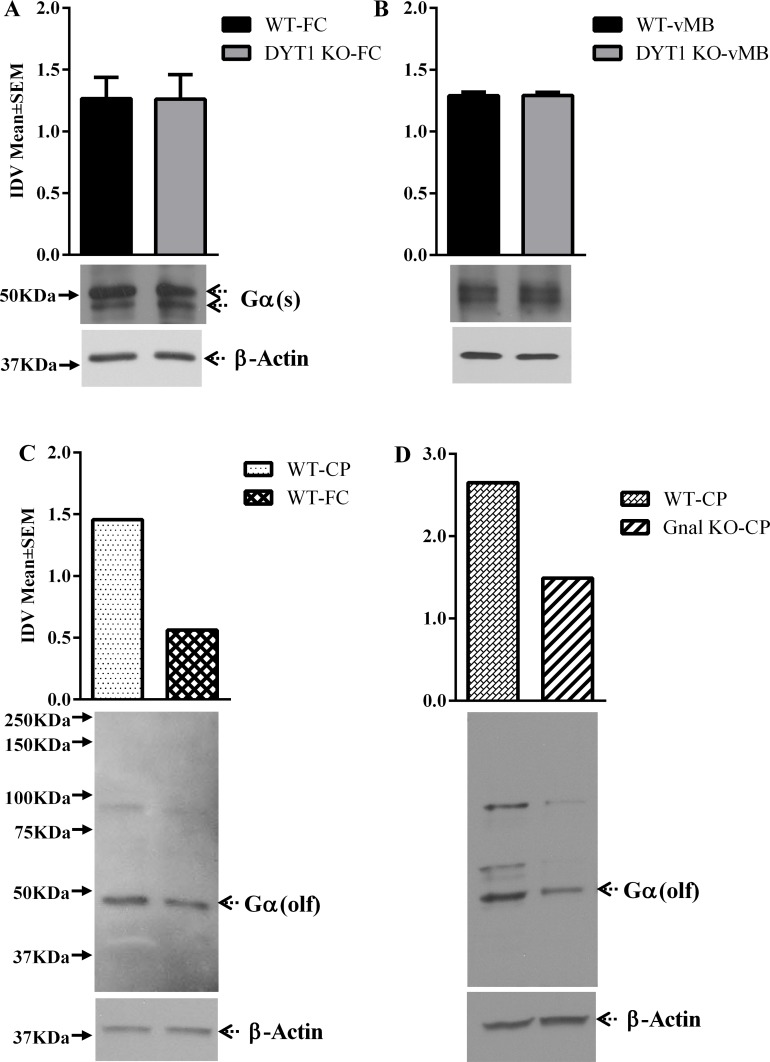
Gα(s) and Gα(olf) expression at P60. Gα(s) and Gα(olf) expression in the frontal cortex, caudate-putamen and ventral midbrain of Dyt1 KO and Gnal KO mice at postnatal day 60 (P60; A-D). In each panel, the upper band shows Gα(s) (Fig 4A and 4B), and Gα(olf) (Fig 4C and 4D), and the lower band shows β-actin, which was used as a loading control. The bar graphs in each panel represent Gα(s), and Gα(olf) band intensity (mean ± SEM) normalized to intensity of loading control (integrated density value; IDV). The names of the mouse lines (*Dyt1* KO, and *Gnal* KO) are indicated to the right.

Specificity of the Gα(olf) antibody was verified by the manufacturer using a peptide containing amino acids 60–90 of human Gα(olf) protein. The peptide used as the immunogen for this antibody has no homology with Gα(s). Since Gα(olf) and Gα(s) share homology, and since Gα(s) is not associated with dystonia, it became necessary to verify that the changes in Gα(olf) reported above were not somehow influenced by potential changes in Gα(s). Toward that end we performed additional studies using a Gα(s) polyclonal antibody (Santa Cruz, sc-823), which recognizes a 15–20 amino acid epitope that maps to a 50 amino acid stretch (aa 100–150) of the Gα(s) protein (accession number P63092). The short and long forms of Gα(s) only differ between each other at residues 71–72 and 73–86. Since this difference lies outside the amino acid sequence used to raise the antibody, the antibody detects both forms of the protein. We examined Gα(s) protein expression in every brain region of the *Dyt1* KO mice in which significant reductions in Gα(olf) protein were found. Our data show that expression levels of neither the short nor the long form of Gα(s) protein were significantly different between the WT and the *Dyt1* KO in any brain region examined [F = (5,12) = 3.099, P > 0.05, Mean± SEM; frontal cortex: WT = 1.27±0.17, KO = 1.26±0.20; ventral midbrain: WT = 1.29±0.03, KO = 1.29±0.03 Fig 4A and 4B].

We performed two additional studies to confirm specificity of the Gα(olf) antibody. First, we compared expression levels of Gα(olf) in the caudate putamen and frontal cortex of wild type P60 mice. Consistent with earlier reports using different Gα(olf) antibodies [36,37], Gα(olf) expression detected by the antibody used here was higher in the caudate putamen compared to the frontal cortex [Fig 4C]. Next, we examined Gα(olf) expression in samples of the caudate putamen obtained from adult heterozygous *Gnal* knockout mice [38]. We found a 56% reduction in Gα(olf) expression in the heterozygous samples [Fig 4D]. Based on these observations we suggest that the Gα(olf) antibody used here is capable of detecting the mouse Gα(olf) protein.

Although the Gα(olf) antibody identified a single prominent 50 KDa band, consistent with the molecular weight of the Gα(olf) protein, in the frontal cortex sample (Fig 4C), additional bands were recognized in the caudate putamen samples (Fig 4C and 4D). Virtually all the bands, not only the 50 KDa band, showed reduced intensity in the caudate putamen of the *Gnal* knockout mouse (Fig 4D). We suggest that the additional bands identified by this antibody represent some form of posttranslational modification of the Gα(olf) protein, especially in the caudate putamen, and that the reduction in the Gα(olf) protein in the *Gnal* knockout mouse not only leads to a decrease in the 50 KDa species but also in the other forms of the Gα(olf) protein.

### D2R expression

**Fig 5:**
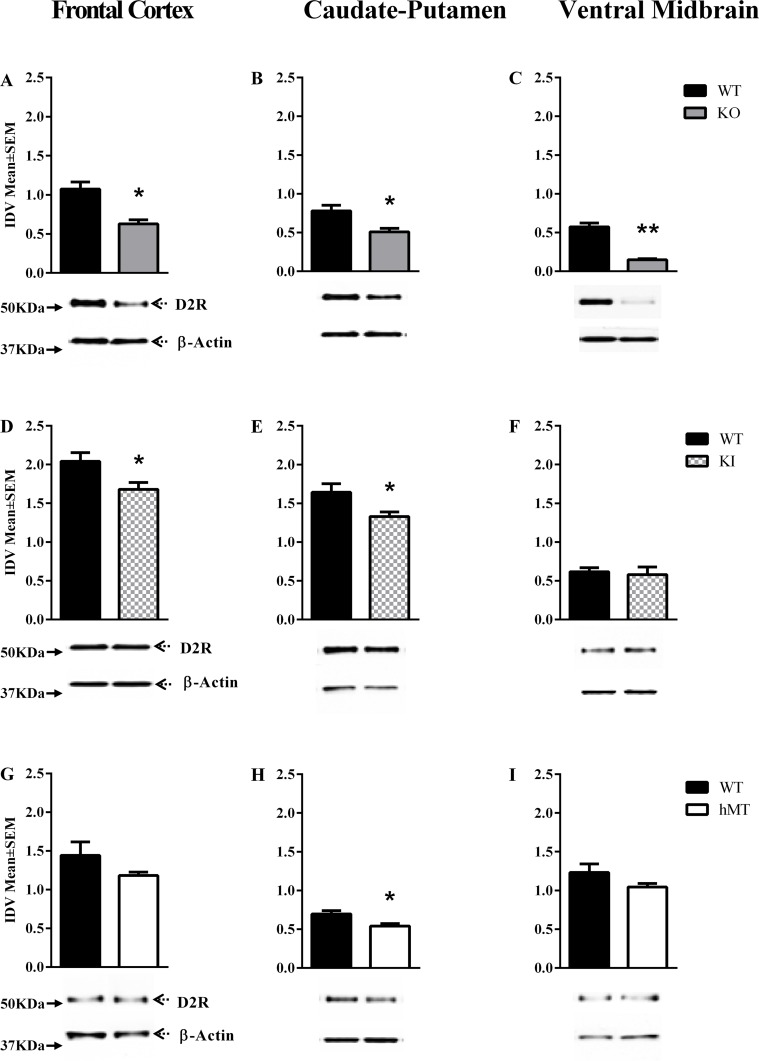
D2R expression at P60. D2R expression in the frontal cortex, caudate-putamen and ventral midbrain of postnatal day 60 (P60; A-I) mice. In each panel, the upper band shows D2R and the lower band shows β-actin, which was used as a loading control. The bar graphs in each panel represent D2R band intensity (mean ± SEM) normalized to intensity of loading control (integrated density value; IDV). The names of the mouse lines (*Dyt1* KO, *Dyt1* KI, hMT) are indicated to the right (*p<0.05, **p<0.01; n = 3 or 4).

D2R expression was significantly altered at P60 [Table 1] in the *Dyt1* KO [F = (5,12) = 26.88, P < 0.0001], and *Dyt1* KI lines (F = (5,18) = 44.60, P < 0.0001). Post hoc analysis indicated significant reductions in the frontal cortex, caudate-putamen and ventral midbrain of the *Dyt1* KO line [Mean± SEM; frontal cortex: WT = 1.08±0.09, KO = 0.63±0.05, p < 0.0005; caudate-putamen: WT = 0.78± 0.07, KO = 0.51± 0.05, p < 0.021; ventral midbrain: WT = 0.57±0.05, KO = 0.15±0.01, p < 0.001; Fig 5A–5C], as well as the frontal cortex,of the *Dyt1* KI line [Mean± SEM; frontal cortex: WT = 2.04± 0.11, KI = 1.68±0.09, p < 0.031 Fig 5D]. Whereas the P60 *DYT1* hMT and hWT mice did not display any significant changes in D2R expression in any brain region [S3 Table] Similarly, we did not find significant changes in D2R expression in any brain region of the *Dyt1* KI or KO mice at P14 [Table 2 and S6 Table].

### Relationship among genotype, developmental stage, brain region and dopamine receptor expression

The use of *Dyt1* KI and *Dyt1* KO lines of mice and the availability of juvenile (P14) and young adult (P60) developmental time periods offered us the opportunity to evaluate the relative impact of the loss of torsinA (*Dyt1* KO) *versus* the presence of the mutant torsinA (*Dyt1* KI), and the impact of developmental stage (P14 *versus* P60) on the expression of D1R and D2R proteins in the frontal cortex and the caudate-putamen. The reductions in D2R protein expression in the frontal cortex and caudate-putamen were greater in magnitude in the *Dyt1* KO mice compared to the *Dyt1* KI mice at P60 [Table 3]. At P14, only the *Dyt1* KO caudate-putamen showed significant changes (reductions) in the D1R protein [Table 3]. Thus, genotype, developmental stage and brain region appeared to impact D1R and D2R expression. Since the hWT and hMT lines were not analyzed at P14, similar comparisons were not performed for those lines.

**Table 3:** A comparison of the magnitude of reductions in D1R and D2R protein expression.

	D1R	D2R
**P60—FC**	No change	KO **>** KI
**P60—CP**	KO **=** KI	KO **>** KI
**P14—FC**	No change	No change
**P14—CP**	KO **>** KI	No change

A comparison of the magnitude of reductions in D1R and D2R protein expression in the frontal cortex (FC) and caudate putamen (CP) of postnatal day 60 (P60) and postnatal day 14 (P14) Dyt1 KO (KO) and Dyt1 KI (KI) mice.

## Discussion

Our data show that dopamine receptor and Gα(olf) protein expression are significantly decreased in multiple brain regions of *Dyt1* KI and *Dyt1* KO mice whereas the expression is not altered in the hMT or hWT mice at P60 [Table 1]. In the juvenile mice (P14) significant decreases occurred in D1R in the caudate-putamen of the *Dyt1* KO mice [Table 2]. Gα(olf) expression did not show significant changes in any brain region in either mouse line at P14 [Table 2]. The ventral midbrain showed significant decreases in the expression of D1R and D2R in the *Dyt1* KO line at P60. Our data demonstrate that impairment of dopaminergic neurotransmission is a common theme in two of the four mouse models of DYT1 dystonia examined here, and that reduction in the expression of D1R but not D2R may begin early in the juvenile period. Interestingly, there was no evidence of significantly increased expression of D1R, D2R or Gα(olf) in any of the regions investigated, at either postnatal age, and in any of the mouse lines examined, indicating that a common feature of the DYT1 phenotype is attenuation, rather than enhancement of dopamine receptor signaling.

Although the effects of the loss of one wild type *Dyt1* allele in the *Dyt1* KO mouse and the introduction of one copy of the ΔGAG allele in the *Dyt1* KI mouse produced comparable effects on D1R and D2R expression at P60 in terms of the direction of the change (decreased expression of both proteins and in both genotypes; Table 1), there were differences between the two mouse lines in terms of the magnitude of the effects [Table 3]. Thus, at P60, the *Dyt1* KO line showed greater decreases compared to the *Dyt1* KI line [Table 3] in D2R expression levels in the frontal cortex. In other words, loss of the wild type torsinA and introduction of the mutant torsinA produced a difference in the magnitude of the effect on D1R and D2R expression at P60. Molecular insights into the mechanisms of such differential effects were not possible. However, both the D1R and D2R mediated pathways, the direct and indirect striatal pathways, respectively may be compromised in DYT1 dystonia, as suggested in earlier reports from other laboratories [39–41]

There were differences in the presence or absence of an effect on D1R expression between *Dyt1* KO and *Dyt1* KI lines in a brain region-dependent manner at P14 [Table 3]. Thus, D1R expression in the caudate-putamen was decreased in the *Dyt1* KO line and was unaffected in the *Dyt1* KI line. Interestingly, D2R expression was unaffected in either mouse line in either brain region at P14 [Table 2]. Thus, loss of the wild type torsinA and introduction of the mutant torsinA produced markedly different effects on dopamine receptor expression at P14, suggesting that the developmental stage is an important variable in modifying the effects of torsinA on dopamine receptor expression.

Here we wish to emphasize two points from the literature. First, a patch-clamp study of the *DYT1* hMT mouse line showed significant impairment of D2R signaling in striatal cholinergic interneurons at P10. Since the cholinergic interneurons constitute a very small proportion of striatal cells (<1% of neurons), it is possible that our western blot data did not detect potential changes in D2R expression in the *DYT1* hMT line or other lines at P14. Second, it would have been interesting to correlate changes in D1R expression at P14 with behavioral changes at early developmental stages in a genotype-phenotype correlation. However, unfortunately, for the mouse lines used in this study phenotype analysis during development (e.g. P14) has not been performed. The only relevant behavioral analysis may be the one in *Dyt1* KI (heterozygous) mice at 3- and 6-months of age, where a deficit in beam walking was noted at 6-months but not at 3-months [20].

That ontogenetic factors may modify torsinA’s effects on dopamine receptor expression is not unexpected. For example, expression of D1R and D2R mRNA and protein, relative distribution of receptor binding sites, and behavioral consequences of receptor activation show brain region-specific ontogenetic changes [42,43]. Although these developmental changes may not fully correlate with one another (e.g. changes in mRNA and protein expression for the same receptor may not correlate with each other), it appears that dopamine receptor expression and function undergo significant region-specific remodeling during development. The cell biological mechanisms underlying the developmental changes likely include mRNA and protein trafficking, membrane recycling and protein conformational changes in response to the changing functional demands on the neurotransmitter signaling machinery of the developing animal. TorsinA is thought to be involved in all these cellular processes [44–46], suggesting impairment of one or more of these processes in our mouse models as a contributor to the decrease in D1R, D2R or Gα(olf) proteins.

We observed a decrease in D1R but not D2R expression at P14, whereas both the receptors were decreased at P60 in multiple brain regions in the KO and KI lines. The relative abundance of the D1R *versus* D2R shows significant ontogenetic changes [25,42,43], which could underlie the differential susceptibilities of the two proteins during the juvenile period to changes in torsinA expression or function. Another consideration is the potential compensatory effects of torsinB, which shares 70% homology with torsinA [6]. We have shown previously that torsinA expression in the brain is high during development and low at maturity, whereas torsinB expression shows the opposite trend (low during development and high at maturity) [24]. However, the expression of torsinB in the mouse models used in the present study is not fully characterized. Therefore, a role for torsinB in the modulation of the effects of torsinA on dopamine receptor protein expression remains speculative.

Introduction of the human ΔGAG allele (hMT) and the human wild type *DYT1* (hWT) produced no significant effects on D1R, D2R or Gα(olf) protein expression in any brain region at P60. Earlier studies using electrophysiological methods showed functional deficits in dopamine D2R in the striatum of the hMT line [40] and behavioral analyses showed motor learning deficits in this mouse line [21]. The lack of significant changes in the expression of any of the proteins in the present study may be due to the global nature of the present analyses (i.e. protein expression analysis rather than functional analysis of the receptors).

Expression of the Gα(olf) protein was significantly decreased in the frontal cortex and ventral midbrain of the *Dyt1* KO line. Recent evidence suggests that mutations in the Gα(olf) gene (GNAL) are associated with generalized dystonia [18]. Gα(olf) is enriched in neurons of the caudate-putamen. However, we did not observe significant changes in Gα(olf) expression in the caudate-putamen in any of the mouse lines.

Finally D1R and D2R expression was affected in the midbrain region only in the *Dyt1* KO line. This may represent an example of region-specificity of the effects of the loss of torsinA. Alternatively, since the expression of D1R and D2R is relatively low in the ventral midbrain compared to the other brain regions, it is possible that our techniques are not sensitive enough to detect additional changes.

In summary our data show that dopamine D1R, D2R and Gα(olf) protein expression is vulnerable to the loss of torsinA as well as introduction of the mouse ΔGAG mutation. The *Dyt1* KO and *Dyt1* KI lines showed the most marked reductions in the expression of the proteins. The decrease in D1R occurred in the juvenile period (P14) and preceded the decreases in D2R and Gα(olf) expression, the latter occurring only at P60. Thus, changes in D1R signaling may occur early in postnatal development predisposing the individual carrying the *Dyt1* mutation to dystonia beginning at an early age. Our findings represent the first comprehensive study of the changes in dopamine receptor signaling mechanisms in DYT1 dystonia.

## Supporting Information

S1 Table D1R expression at P60. (DOCX)

S2 Table Gα(olf) expression at P60. (DOCX)

S3 Table D2R expression at P60. (DOCX)

S4 Table D1R expression at P14. (DOCX)

S5 Table Gα(olf) expression at P14. (DOCX)

S6 Table D2R expression at P14. (DOCX)
