# Supplementary material for: Dopamine Receptor and Gα(olf) Expression in DYT1 Dystonia Mouse Models during Postnatal Development
Source: PLoS One. 2015 Apr 10;10(4):e0123104. doi: 10.1371/journal.pone.0123104 (PMC4393110; doi:10.1371/journal.pone.0123104)
Supplement: S3 Table — (DOCX) [file pone.0123104.s003.docx]

**S3 Table. D2R expression in P60 mice.**

|  | **WT** | **hWT** |
| --- | --- | --- |
|  | Mean±SEM | Mean±SEM |
| FC | 2.11 ± 0.19 | 2.50 ± 0.18 |
| CP | 0.81 ± 0.01 | 0.79 ± 0.04 |
| vMB | 1.93 ± 0.33 | 1.92 ± 0.29 |
|  |  |  |
|  | **WT** | **hMT** |
|  | Mean±SEM | Mean±SEM |
| FC | 1.44 ± 0.17 | 1.18 ± 0.05 |
| CP | 0.7 ± 0.15 | 0.54 ± 0.01 |
| vMB | 1.23 ± 0.11 | 1.04 ± 0.05 |
|  |  |  |
|  | **WT** | **KI** |
|  | Mean±SEM | Mean±SEM |
| CP | 0.70 ± 0.05 | 0.54 ± 0.03 |
| vMB | 0.62 ± 0.05 | 0.58 ± 0.09 |

Mean±SEM values for D2R expression in the frontal cortex (FC), caudate putamen (CP) and ventral midbrain (vMB) at postnatal day 60 (P60) where the differences between wild type (WT) and transgenic (hWT, hMT or KI) littermates within a given line did not reach statistical significance (One-way ANOVA; p>0.05).
