# Supplementary material for: Dopamine Receptor and Gα(olf) Expression in DYT1 Dystonia Mouse Models during Postnatal Development
Source: PLoS One. 2015 Apr 10;10(4):e0123104. doi: 10.1371/journal.pone.0123104 (PMC4393110; doi:10.1371/journal.pone.0123104)
Supplement: S4 Table — (DOCX) [file pone.0123104.s004.docx]

**S4 Table. D1R expression in P14 mice.**

|  | **WT** | **KI** |
| --- | --- | --- |
|  | Mean±SEM | Mean±SEM |
| FC | 1.43±0.09 | 1.42±0.07 |
| CP | 0.78±002 | 0.62±0.02 |
|  |  |  |
|  | **WT** | **KO** |
|  | Mean±SEM | Mean±SEM |
| FC | 0.96±0.01 | 0.79±0.04 |

Mean±SEM values for D1R expression in the frontal cortex (FC) and caudate putamen (CP) at postnatal day 14 (P14) in wild type (WT) and transgenic (KI and KO) littermates where the differences did not reach statistical significance (One-way ANOVA; p>0.05).
